# Enhanced serodiagnosis of opisthorchiasis using a multi-epitope dot-ELISA: comparative evaluation of visual and imageJ-assisted analysis of IgG and IgM responses

**DOI:** 10.1186/s12879-026-13445-w

**Published:** 2026-04-29

**Authors:** Jittiyawadee Sripa, Phalakorn Suebsamran, Kanjana Pangjit

**Affiliations:** 1 College of Medicine and Public Health, Ubon Ratchathani University, Warinchamrap, Ubon Ratchathani, 34190 Thailand; 2 Research Group for Biomedical Research and Innovative Development (RG- BRID), College of Medicine and Public Health, Ubon Ratchathani University, Warinchamrap, Ubon Ratchathani, 34190 Thailand

**Keywords:** Opisthorchiasis diagnosis, Dot-ELISA, Multi-epitope recombinant antigen, IgG and IgM detection, ImageJ-assisted analysis

## Abstract

**Background:**

Opisthorchiasis remains an important public health problem in Southeast Asia and is strongly associated with cholangiocarcinoma. Although stool-based diagnosis is widely used, its sensitivity is limited in light infections, highlighting the need for alternative diagnostic approaches. This study developed and evaluated a dot-enzyme-linked immunosorbent assay (dot-ELISA) based on a multi-epitope recombinant antigen (OvCB_OvAEP_OvCF) for the serological detection of *Opisthorchis viverrini*, and compared conventional visual interpretation with ImageJ-assisted digital analysis for IgG and IgM antibody detection.

**Methods:**

The assay was optimised using pooled positive and negative sera, and cut-off values for ImageJ-based interpretation were determined by receiver operating characteristic (ROC) analysis. Diagnostic performance was evaluated using 70 serum samples, including sera from patients with confirmed opisthorchiasis, other parasitic infections, and negative controls. Diagnostic performance parameters were calculated. Agreement between methods was assessed using Cohen’s kappa, while paired differences were evaluated using McNemar’s test and the Wilcoxon signed-rank test. Logistic regression analysis was performed to identify predictors of infection. The stability of antigen-coated nitrocellulose membranes was assessed under different storage conditions.

**Results:**

Visual interpretation of the optimised dot-ELISA yielded high sensitivity for both IgG (93.33%) and IgM (100%) detection, although specificity was limited, particularly because of cross-reactivity with other parasitic infections. ImageJ-assisted analysis improved specificity for IgG (76.36%) and IgM (45.45%) detection and increased diagnostic accuracy for both IgG (74.29%) and IgM (55.71%). ROC analysis showed moderate discriminatory ability for IgM detection (AUC = 0.797, *P* < 0.001), whereas IgG detection showed limited discrimination (AUC = 0.578, *P* = 0.458). Agreement analysis showed substantial concordance for IgM detection and moderate agreement for IgG detection. Paired statistical analyses revealed significant differences between visual and ImageJ-based interpretations, indicating that digital quantification altered classification outcomes and improved discrimination between antibody responses. Logistic regression identified IgM detection as the strongest predictor of infection. The recombinant antigen remained stable on nitrocellulose membranes for up to three months for IgG detection and up to two months for IgM detection under all tested storage conditions.

**Conclusions:**

The developed dot-ELISA platform showed promising potential as a serological screening tool for opisthorchiasis, particularly for IgM antibody detection. ImageJ-assisted analysis improved the objectivity, reproducibility and diagnostic discrimination by reducing observer bias. Furthermore, the stability of the antigen-coated membranes supports the feasibility of field-based application. Although specificity requires further improvement, this platform may provide a practical and scalable approach for screening and surveillance of opisthorchiasis in endemic and resource-limited settings.

**Supplementary Information:**

The online version contains supplementary material available at 10.1186/s12879-026-13445-w.

## Background

Opisthorchiasis, caused by the liver fluke *Opisthorchis viverrini* (OV), remains a major public health concern in Southeast Asia and is strongly associated with the development of cholangiocarcinoma (CCA). Early detection of infection is therefore essential for effective disease control and for preventing long-term complications. Traditionally, the diagnosis of opisthorchiasis relies on the microscopic detection of OV eggs in faecal samples. The microscopic examinations, such as the modified formalin–ether concentration technique (m-FECT) and the Kato–Katz thick smear, are widely used to improve the sensitivity of egg detection [1]. However, stool examination has several limitations. Egg output can vary considerably, particularly in light infections, which may lead to false-negative results. In addition, reliable detection often requires examination of multiple stool samples and repeated microscopic observations, making the procedure labour-intensive and inconvenient for large-scale screening programmes [2].

To overcome these limitations, a range of immunological and molecular diagnostic approaches has been developed. Serological assays, including enzyme-linked immunosorbent assay (ELISA) and immunoblotting, detect host antibodies against parasite antigens and demonstrate improved sensitivity over stool microscopy while enabling high-throughput analysis of multiple samples [3, 4]. Molecular techniques, particularly polymerase chain reaction (PCR) and loop-mediated isothermal amplification (LAMP), allow sensitive detection of OV DNA in stool, even in low-intensity infections [5, 6]. However, LAMP is prone to non-specific amplification due to the complexity of primer design, which may result in false-positive findings, especially when visual detection methods are applied [7]. In addition, its qualitative nature and susceptibility to carry-over contamination may limit analytical reliability and reproducibility [8]. Despite these advances, both immunological and molecular approaches generally require specialised laboratory infrastructure, trained personnel, and relatively high operational costs, thereby limiting their scalability in endemic and resource-limited settings [9].

Dot-ELISA represents a simplified immunoassay format that shares methodological principles with conventional ELISA and immunoblotting but requires minimal instrumentation. In this technique, antigens are immobilised on nitrocellulose membranes (NC), and antigen–antibody reactions are visualised as coloured dots following an enzymatic reaction. Dot-ELISA offers several practical advantages, including reduced reagent consumption, rapid assay procedures, minimal equipment requirements, and the possibility of visual interpretation without specialised laboratory instruments [10]. These characteristics make dot-ELISA particularly attractive for diagnostic applications in resource-limited settings and for large-scale epidemiological investigations. However, the interpretation of dot-based assays is commonly based on visual assessment of signal intensity, which may introduce subjectivity, particularly when colour differences between positive and negative reactions are subtle.

Recent advances in digital image analysis have created opportunities to improve the interpretation of dot-based immunoassays. ImageJ, a widely used open-source image-processing software developed by the National Institutes of Health (NIH), enables objective measurement of signal intensity from digital images [11]. The integration of ImageJ-assisted analysis with dot-ELISA allows semi-quantitative evaluation of immunoassay signals, potentially reducing observer bias and improving the reproducibility of diagnostic interpretation. Furthermore, the dot-based format allows assay results to be easily captured using digital imaging devices such as smartphone cameras, facilitating image archiving, remote consultation, and potential tele-diagnostic applications. However, the comparative performance of conventional visual inspection and ImageJ-assisted analysis in the context of opisthorchiasis diagnosis remains insufficiently explored.

In addition to methodological considerations, the selection of appropriate antibody targets is critical for improving diagnostic performance. IgM and IgG antibodies play distinct roles in the host immune response to parasitic infection. IgM antibodies are typically associated with early or recent infection, whereas IgG antibodies may persist for extended periods and reflect both past and current exposure. Comparative evaluation of IgM and IgG responses may therefore provide additional diagnostic value by improving the ability to distinguish between different stages of infection and enhancing overall diagnostic discrimination. Nevertheless, few studies have systematically compared these antibody classes in dot-ELISA-based detection of OV.

Parasite-derived proteolytic enzymes have been identified as promising antigenic targets for the serodiagnosis of opisthorchiasis. Antibodies against OV proteases, including cathepsin B (Ov-CB-1) [4], cathepsin F (Ov-CF) [12], and asparaginyl endopeptidase (Ov-AEP) [3], have previously been detected using ELISA and immunoblotting techniques. Based on these findings, highly antigenic B-cell epitopes derived from these enzymes were combined to generate a multi-epitope recombinant protein (OvCB_OvAEP_OvCF). This recombinant antigen has previously demonstrated excellent sensitivity (100%) for detecting opisthorchiasis, particularly in light-infection cases when evaluated using immunoblotting, indicating its strong potential as a diagnostic antigen [13].

Therefore, the present study aimed to develop and evaluate a dot-ELISA platform using the multi-epitope recombinant antigen OvCB_OvAEP_OvCF for the serodiagnosis of opisthorchiasis. The study further assessed the detection of both IgG and IgM antibodies in human sera in order to improve diagnostic sensitivity and accuracy. In addition, conventional visual interpretation was compared with ImageJ-assisted digital analysis to determine whether image-based quantification could enhance diagnostic interpretation and reproducibility. Finally, the stability of the OvCB_OvAEP_OvCF protein immobilised on NC under different storage temperatures and durations was evaluated to determine the robustness and shelf life of the dot-ELISA platform and to assess its feasibility for application in field-based diagnostic settings. By integrating recombinant antigen design with digital image analysis, this study aims to contribute to the development of more accessible, reliable, and scalable diagnostic tools for opisthorchiasis, particularly in endemic and resource-limited settings.

## Materials and methods

### Clinical human sera

Frozen sera from the frozen bank of the College of Medicine and Public Health, Ubon Ratchathani University, were utilised to optimise and evaluate the performance of the in-house dot-ELISA in diagnosing opisthorchiasis. A total of 30 frozen serum samples, comprising 15 negative parasite infections and 15 opisthorchiasis sera, were employed to establish the optimal cut-off for dot-ELISA. Additionally, 70 frozen sera were analysed, categorized as negative parasite infections (*n* = 15), opisthorchiasis (*n* = 15), strongyloidiasis (*n* = 13), taeniasis (*n* = 10), *Entamoeba coli* infections (*n* = 4), giardiasis (*n* = 4), hookworm infection (*n* = 3), enterobiasis (*n* = 1), trichuriasis (*n* = 1), mixed infection of strongyloidiasis and hookworm infection (*n* = 3), and mixed infection of taeniasis and echinostomiasis (*n* = 1) to assess the efficacy of dot-ELISA in diagnosing opisthorchiasis. The helminth infection sera utilised in this study, including opisthorchiasis, hookworm infection, enterobiasis, trichuriasis, and echinostomiasis, represented light-infected cases.

### Recombinant protein preparation

A clone of the multi-epitope recombinant DNA, OvCB_OvAEP_OvCF_pET32a+, obtained from a previous study [13], was expressed as a recombinant protein in a bacterial system. The OvCB_OvAEP_OvCF clone in pET32a + was cultured in *Escherichia coli* strain BL21(DE3) overnight (16–18 h) at 37 °C in 50 mL Luria–Bertani (LB) broth containing 100 µg/mL ampicillin and agitation at 200 rpm. The bacterial pellet was collected as the starter for further protein expression induction in 200 mL fresh LB broth with 100 µg/mL ampicillin and 1 mM IPTG. After induction of the protein expression for 8–10 h, at 37 °C with agitation at 200 rpm, the bacterial pellet was harvested and lysed in denaturing binding buffer (5 mM imidazole, 0.5 M NaCl, 20 mM Tris–HCl, 8 M urea, pH 7.9) through freeze-thaw and sonication on ice (4 °C) at 25% amplitude for 5 min with a pulse on and off every 5 s (ultrasonic sonicator, VCX 750 W, Sonics, USA). The bacterial debris was then removed, and the supernatant was collected for further purification.

The supernatant containing the desired multi-antigenic recombinant protein was purified through a Ni-NTA column (HisPur™ Ni-NTA resin, Thermo Scientific™, USA) under denaturing conditions. The purified protein was analysed using 15% SDS-PAGE, and its concentration was measured with a NanoDrop Microvolume Spectrophotometer (Thermo Fisher Scientific, USA). The purified recombinant protein was aliquoted at 1 mg/mL and stored at -20 °C for further analysis.

### Optimisation of dot-ELISA

Standardisation for dot-ELISA was conducted at room temperature (25 °C) to optimise the concentrations of recombinant protein, serum sample dilutions, and anti-human IgG and IgM horseradish peroxidase (HRP)-conjugates. Initially, NC membrane with a pore size of 0.45 μm (Thermo Fisher Scientific, USA) was cut into strips measuring 2 cm in width and 5 cm in length, which were then placed into the chamber. The denatured form of the recombinant protein was spotted on the strips at concentrations of 50, 100, 500, and 1,000 ng, followed by drying for 1 h. Subsequently, nonspecific binding sites on the strips were blocked by incubating them in 5% BSA-PBST for 1 h. The strips were then washed three times for 3 min each with PBST and incubated in pooled positive opisthorchiasis sera (10-positive opisthorchiasis sera) and pooled negative control sera (10-negative control sera) at dilutions of 1:500, 1:1,000, 1:2,000, and 1:4,000 in 5% BSA-PBST for 1 h. Following this incubation, the strips were washed thrice for 3 min each with PBST. The bound human IgG and IgM antibodies were detected using goat anti-human IgG and IgM antibody HRP conjugates (Rockland, USA) at dilutions of 1:2,500 and 1:5,000 in 5% BSA-PBST for 1 h. Following the incubation, the strips were washed twice, 3 min each, with PBST and PBS for 3 min. Subsequently, the strips were soaked in 3,3’,5,5’-tetramethyl benzidine (TMB)-Blotting Substrate Solution (Thermo Scientific™, Thermo Fisher Scientific, USA) for 1 min. The reaction was halted by washing the strips with tap water, and the strips were then dried. The reactivity, indicated by blue spots, was observed through visual inspection.

The raw integrated colour density was chosen as the main metric for ImageJ-based quantification of dot-ELISA signals. The dot-ELISA image was captured at a distance of 10–15 cm vertically from the NC position, without a flashlight, using a consistent brightness level of 20%, determined with a light meter. The image was then analysed for the raw integrated colour density of each dot by subtracting the background, following the ImageJ tutorial for dot blot analysis available at https://imagej.net/ij/docs/examples/dot-blot/ (accessed 13 July 2025). In short, the dot-ELISA image was opened using ImageJ software (NIH ImageJ software, version 1.54p) [11]. The image was resized to 50 pixels in width and height, and the image depth was adjusted to 1. Subsequently, the image was inverted to eliminate background noise. In the background-subtracted window, the image pixel value was set to 50 pixels, and the preview option was chosen to verify background subtraction. The parameters of area and integrated density were chosen for quantification. The measurement results, including area, internal density (integrated density), and raw internal density (raw integrated density), were presented in the result window. Once all measurements were completed, they were transferred to Microsoft Excel for further analysis.

The optimal conditions for dot-ELISA were established by identifying the point with the most significant contrast in chromogenic intensity between pooled positive and negative sera against a clear background. These conditions were subsequently applied to determine the cut-off value of the dot-ELISA and analyse the serum samples.

### Determination of the cut-off values of dot-ELISA

The cut-off values for IgG and IgM detection were determined using the optimised dot-ELISA. In brief, the NC was cut into squares (1 cm wide and 1 cm long) and placed in 24-well microplates. The optimal concentration of recombinant protein for detecting IgG and IgM antibodies was dotted on the NC and air-dried at room temperature (25 °C) for 1 h. Subsequently, nonspecific binding sites on each membrane were blocked with 400 µL of 5% BSA-PBST for 1 h, followed by three washes, each lasting 3 min, with 500 µL of PBST. Afterwards, each membrane was incubated with 400 µL of the optimal dilution of human sera, including 15 positive and 15 negative control sera for OV, for 1 h. Following the washing step, 400 µL of the optimal dilution of secondary antibody-HRP conjugates was incubated with the membrane for 1 h. The colour development was then carried out after washing twice with 500 µL of PBST for 3 min each and 500 µL of PBS for 3 min, by adding 300 µL of TMB (Thermo Scientific™, ThermoFisher Scientific, USA) to determine reactivity through visual inspection. The raw integrated colour intensity of the blue spot was quantified using ImageJ as described previously.

The raw integrated colour intensity of each dot was utilised to create receiver operating characteristic (ROC) curves using MedCalc^®^ Statistical Software version 23.1.3 (MedCalc Software Ltd, Ostend, Belgium; https://www.medcalc.org; 2025) to calculate the area under the curve (AUC). The cut-off value that produced the highest accuracy was identified using Youden’s index.

### Determining the stability of the recombinant protein coated on the NC membrane

The NC was cut into squares (1 cm wide and 1 cm long) and placed in 24-well microplates. The recombinant protein, at concentrations of 50 and 100 ng/µL, obtained from the optimisation step, was dotted on the NC. After drying for 1 h at room temperature (25 °C), these recombinant protein-coated NC were stored at room temperature (25 °C), 4, −20, and −80 °C for 1, 2, and 3 months to examine the stability of the recombinant protein in the detection of IgG and IgM antibodies from pool positive and negative control sera for OV. Positive and negative reactivities were determined by visual inspection and ImageJ software. The raw integrated colour intensity of the positive and negative dots at each storage temperature and time was compared with the initial intensity at the first time (day 1, D1).

### Performance evaluation of dot-ELISA

The diagnostic performance of the dot-ELISA for the detection of opisthorchiasis was evaluated using two interpretation approaches: visual inspection and ImageJ-assisted digital analysis. Dot-ELISA membranes were photographed under standardised lighting conditions using a fixed imaging setup to ensure consistent image acquisition and minimise variation in signal intensity caused by environmental factors.

For visual interpretation, the developed dots were examined independently and categorised as either positive (+) or negative (−) based on visible colour development. Weakly positive dots were considered positive.

For digital interpretation, the captured images were analysed using ImageJ software (NIH ImageJ software, version 1.54p) [11]. The signal intensity of each dot was quantified using the raw integrated density measurement after background subtraction. An optimal cut-off value for IgG and IgM detection obtained from ROC curve described previously was used to classify results as positive (+) or negative (−).

### Statistical analysis

Statistical analyses were performed to evaluate diagnostic performance and to compare interpretation methods and antibody classes. Sensitivity, specificity, positive predictive value (PPV), negative predictive value (NPV), and diagnostic accuracy were calculated for each interpretation method and antibody class, using m-FECT as the reference standard.

ROC curve analysis was performed to determine the diagnostic performance of IgG and IgM antibody detection and to identify optimal cut-off values for ImageJ-based signal intensity, which were further used in the classification of samples as positive or negative. The AUC was calculated to assess the overall diagnostic discrimination of each assay. ROC curves were compared using DeLong’s test.

Agreement between visual inspection and ImageJ-assisted interpretation for IgG and IgM assays was evaluated using Cohen’s Kappa (κ) statistic. The level of agreement was interpreted according to the Landis and Koch criteria as follows: <0 = poor, 0.00–0.20 = slight, 0.21–0.40 = fair, 0.41–0.60 = moderate, 0.61–0.80 = substantial, and 0.81–1.00 = almost perfect agreement [14].

McNemar’s test was applied to compare paired diagnostic outcomes between visual inspection and ImageJ-assisted interpretation, as well as between IgG and IgM detection results. The Wilcoxon signed-rank test was used to assess paired differences between related interpretation methods and antibody classes, including comparisons between visual inspection and ImageJ-assisted analysis for IgG and IgM detection, and between IgG and IgM responses within the same interpretation approach. This non-parametric test was selected for paired data that were not assumed to be normally distributed. Hodges–Lehmann median differences and 95% confidence intervals were also calculated. All tests were two-tailed, and a P-value < 0.05 was considered statistically significant.

Binary logistic regression analysis was performed to evaluate the predictive value of antibody detection (IgG or IgM) and interpretation method (visual inspection or ImageJ analysis) for identifying opisthorchiasis. Model fit was assessed using the Hosmer–Lemeshow goodness-of-fit test, and the discriminative ability of the model was evaluated using ROC curve analysis. All statistical tests were two-tailed, and a P-value < 0.05 was considered statistically significant. All statistical analyses in this study were performed using MedCalc^®^ Statistical Software version 23.1.3 (MedCalc Software Ltd, Ostend, Belgium; https://www.medcalc.org; 2025).

## Results

### Optimal conditions and cut-off values for dot-ELISA in diagnosing opisthorchiasis

**Fig. 1:**
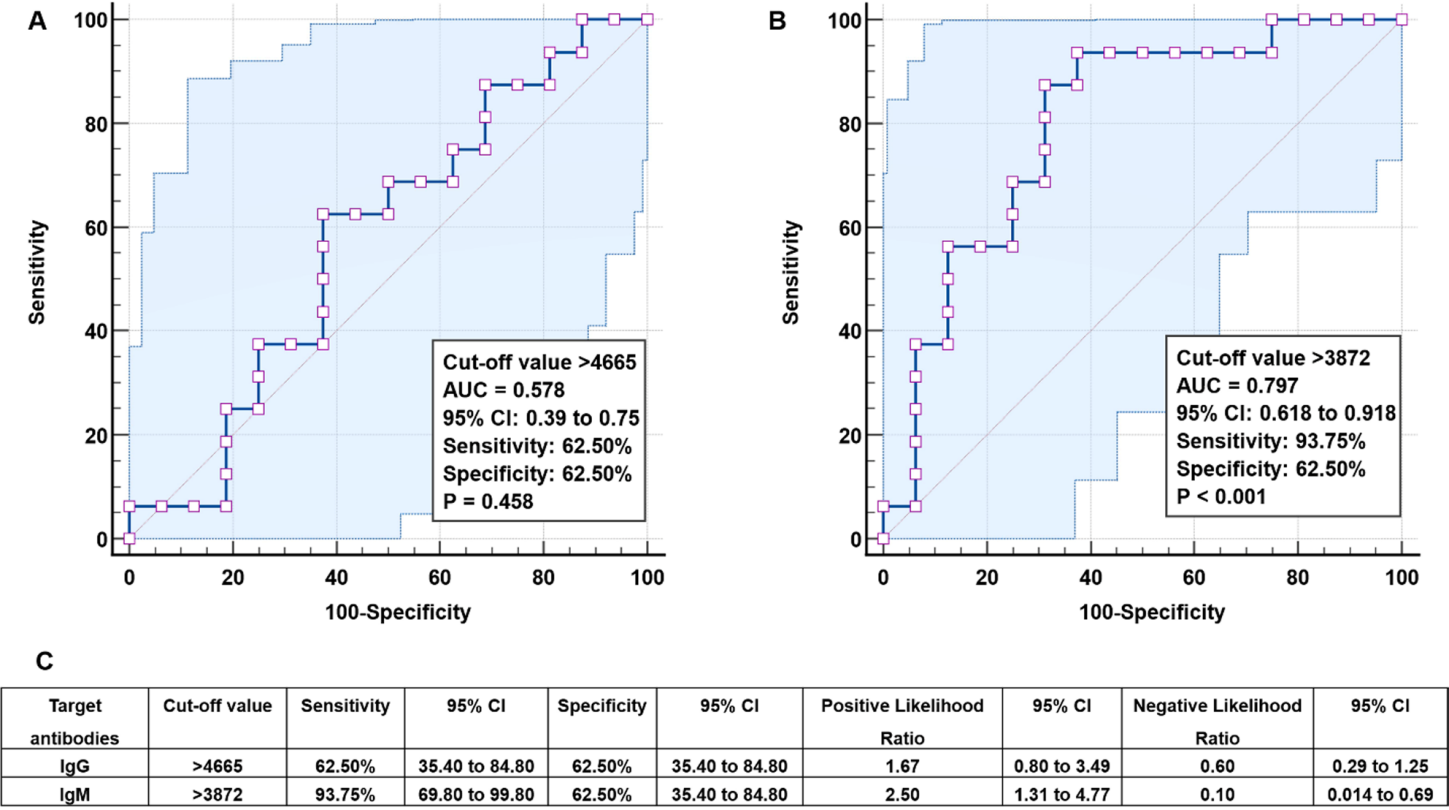
ROC curve analysis for determining optimal cut-off values for IgG and IgM antibody detection using ImageJ-assisted quantification. ROC curves were generated using raw integrated density values derived from ImageJ analysis of dot-ELISA signals to assess the diagnostic performance of IgG (**A**) and IgM (**B**) antibody detection. Optimal cut-off values for classifying positive and negative results were determined using Youden’s index. IgM detection demonstrated higher discriminatory performance compared with IgG, as reflected by the corresponding area under the curve (AUC). Diagnostic performance parameters derived from the ROC-based cut-off values are summarised for each assay (**C**)

The optimal conditions for the dot-ELISA were validated using a pool of sera that were positive and negative for OV. The optimal concentration of recombinant protein at a concentration of 50 ng/µL, the dilutions of human serum of 1:2,000, and the dilution of goat anti-human IgG-HRP antibody of 1:5,000 were chosen for detecting IgG antibody (Figure S1). Using these parameters, the cut-off value for IgG antibody detection was examined using 15 positive and 15 negative serum samples for OV. A raw integrated colour density of > 4665 was chosen as the cut-off to differentiate between positive and negative results. At this cut-off value, an optimal sensitivity of 62.50% and specificity of 62.50% were obtained for diagnosing opisthorchiasis (Fig. 1).

In the detection of IgM antibodies, we selected the optimal concentration of recombinant protein at 100 ng/µL, human serum dilutions of 1:1,000, and goat anti-human IgM-HRP antibody dilution of 1:5,000 (Figure S1). These conditions yielded a cut-off value of the raw integrated colour density > 3872, which provided the highest sensitivity (93.75%) and specificity (62.50%) for opisthorchiasis detection (Fig. 1).

### Performance of the optimised dot-ELISA for diagnosing opisthorchiasis

Seventy human serum samples, including those from healthy individuals, patients with opisthorchiasis, and patients with other parasitic diseases, were utilised to examine the efficacy of the optimised dot-ELISA in diagnosing opisthorchiasis. The optimised dot-ELISA demonstrated high sensitivity in detecting IgG (93.33%) and IgM (100%) antibodies through visual inspection. However, the method exhibited relatively low specificity, with a notable occurrence of false positives in non-opisthorchiasis samples, particularly for IgG (41.82%) and IgM (32.73%) antibody detection by visual inspection. The most prevalent cross-reactivity was observed in the sera of patients with strongyloidiasis, taeniasis, or giardiasis.

The distinction between positive and negative results using ImageJ software proved more effective than visual examination in identifying IgG (76.36%) and IgM (45.45%) antibodies. The software aided in enhancing the analysis of IgG (accuracy = 74.29%) and IgM (accuracy = 55.71%) detection, indicating that digital quantification reduced subjectivity and enhanced diagnostic precision. However, the overall results demonstrated that the ImageJ software remained superior, delivering more reliable and precise results.

Additionally, the positive predictive values (PPV) derived from this test were moderate, indicating that positive results alone may not be definitive without additional confirmatory testing. Furthermore, the negative predictive values (NPV) were outstanding, particularly for the visual inspection of IgM antibodies (100%), indicating strong reliability in excluding infection when the outcomes are negative. Nevertheless, due to the moderate PPV and low specificity, there may be a requirement for further enhancement of antigen selection, testing procedures, or methodologies to reduce potential cross-reactivity with other parasitic infections (Figure S2 and S3, Table 1).

**Table 1:** Diagnostic performance of dot-ELISA-based IgG and IgM antibody detection using optimised cut-off values derived from ROC analysis. Performance metrics, including sensitivity, specificity, likelihood ratios, predictive values, and overall accuracy, are shown with 95% confidence intervals (95% CI). All diagnostic parameters were calculated using 70 proven clinical samples, and the results from visual inspection and ImageJ-assisted quantification for both IgG and IgM detection were compared

Statistical parameters	Detection of IgG antibody				Detection of IgM antibody			
	Visual inspection		NIH ImageJ program		Visual inspection		NIH ImageJ program	
	Value	95% CI	Value	95% CI	Value	95% CI	Value	95% CI
Sensitivity	93.33%	68.05 to 99.83	66.67%	38.38 to 88.18	100.00%	78.20 to 100.00	93.33%	68.05 to 99.83
Specificity	41.82%	28.65 to 55.89	76.36%	62.98 to 86.77	32.73%	20.68 to 46.71	45.45%	31.97 to 59.45
Positive Likelihood Ratio	1.60	1.23 to 2.08	2.82	1.56 to 5.11	1.49	1.24 to 1.79	1.71	1.30 to 2.26
Negative Likelihood Ratio	0.16	0.02 to 1.09	0.44	0.21 to 0.91	0		0.15	0.02 to 1.00
Positive Predictive Value	30.43%	25.19 to 36.24	43.48%	29.79 to 58.23	28.85%	25.21 to 32.77	31.82%	26.14 to 38.09
Negative Predictive Value	95.83%	77.14 to 99.37	89.36%	80.18 to 94.58	100.00%	81.47 to 100.00	96.15%	78.64 to 99.41
Accuracy	52.86%	40.55 to 64.91	74.29%	62.44 to 83.99	47.14%	35.09 to 59.45	55.71%	43.34 to 67.59

ROC curve analysis was performed to evaluate the diagnostic performance of IgG and IgM antibody detection using the ImageJ assisted. For IgG antibody detection, the ROC curve demonstrated limited diagnostic discrimination, with an AUC of 0.578, which was not statistically different from random classification (*P* = 0.458). This result indicates that IgG detection alone had limited ability to distinguish opisthorchiasis from non-opisthorchiasis samples. In contrast, IgM antibody detection showed improved diagnostic discrimination. ROC curve demonstrated an AUC of 0.787 (*p* <0.001), indicating moderate discriminatory ability for identifying opisthorchiasis (Fig. 1).

Direct comparison of ROC curves between IgG and IgM detection using ImageJ programme revealed no statistically significant difference in diagnostic performance (z = − 1.636, *P* = 0.1019). Although IgM detection showed a higher AUC than IgG, the difference did not reach statistical significance. These findings suggest that IgM detection may provide better diagnostic discrimination than IgG detection in the dot-ELISA system using ImageJ programme; however, both antibody classes provide comparable diagnostic discrimination for the detection of opisthorchiasis (Table 2, Table S1).

**Table 2:** Integrated statistical analysis of diagnostic performance and agreement of the dot-ELISA platform. This table summarises the results of multiple statistical analyses, including ROC comparison, Cohen’s kappa, McNemar’s test, Wilcoxon signed-rank test, and logistic regression. ImageJ-assisted analysis improved diagnostic discrimination by reducing misclassification and enhancing agreement, particularly for IgM detection. Wilcoxon and McNemar analyses demonstrated that visual interpretation may overestimate positivity, while logistic regression identified IgM detection as the strongest predictor of infection. Full statistical outputs were provided in the supplementary tables

Analysis	Comparison	Key result	*P*-value	Interpretation
ROC comparison	IgG vs. IgM (ImageJ)	ΔAUC = − 0.219	0.1019	IgM showed higher AUC, but not statistically significant
Cohen’s kappa	IgG Visual vs. ImageJ	κ = 0.449	–	Moderate agreement
	IgM Visual vs. ImageJ	κ = 0.711	–	Substantial agreement
	IgG vs. IgM (Visual)	κ = 0.038	–	Slight agreement
	IgG vs. IgM (ImageJ)	κ = 0.014	–	Slight agreement
McNemar’s test	IgG Visual vs. ImageJ	Δ = 0.300	< 0.0001	Significant difference; reduced misclassification with ImageJ
	IgM Visual vs. ImageJ	Δ = 0.1286	0.0039	Significant difference
	IgG vs. IgM (Visual)	Δ = 0.100	0.2649	Not significant
	IgG vs. IgM (ImageJ)	Δ = 0.2714	0.0026	Significant difference
Wilcoxon signed-rank	IgG ImageJ vs. Visual	Z = − 4.58	< 0.0001	ImageJ reduces overestimation of positivity
	IgM ImageJ vs. Visual	Z = − 3.00	0.0027	Improved classification consistency
	IgG vs. IgM (ImageJ)	Z = − 3.12	0.0018	IgM shows stronger reactivity
	IgG vs. IgM (Visual)	Z = − 1.30	0.1936	No significant difference
Logistic regression	IgM_ImageJ	OR = 24.67	0.006	Strongest predictor of infection
	IgG_ImageJ	OR = 2.74	0.266	Not significant
Model performance	IgM model	AUC = 0.832	0.0001	Good discrimination
	Combined ImageJ model	AUC = 0.842	0.001	Improved performance
	Combined Visual model	AUC = 0.895	< 0.0001	High performance (but potential overfitting)

Agreement between visual inspection and ImageJ-assisted interpretation was evaluated using Cohen’s Kappa statistics. Substantial agreement was observed for IgM detection (κ = 0.711), whereas moderate agreement was found for IgG detection (κ = 0.449). These findings suggest that ImageJ analysis contributed to improved reproducibility and reduced observer bias, particularly in borderline cases. However, cross-assay concordance between IgG and IgM under the same interpretation mode was minimal (κ ≈ 0.02–0.04), aligning with their distinct immunological functions, where IgM signifies early infection, and IgG signifies chronic or previous exposure. These results validate that ImageJ quantification improves the reproducibility and diagnostic accuracy of dot-ELISA-based serodiagnosis of opisthorchiasis (Table 2, Table S2).

Paired comparison using McNemar’s test revealed significant differences between visual inspection and ImageJ-assisted interpretation for both IgG (*P* < 0.0001) and IgM detection (*P* = 0.0039), indicating that digital image analysis significantly altered the classification of samples compared with visual interpretation. When antibody classes were compared, no significant difference between IgG and IgM detection was observed using visual interpretation (*P* = 0.2649). However, a significant difference was observed when ImageJ-assisted analysis was applied (*P* = 0.0026), suggesting that digital quantification enhances the discrimination between antibody responses (Table 2, Table S3).

Wilcoxon signed-rank test analysis revealed significant differences between interpretation methods and antibody classes. For IgG detection, ImageJ-assisted analysis produced significantly different results compared with visual inspection (Z = − 4.58, *P* < 0.0001), indicating that visual interpretation tended to classify more samples as positive. A similar but less pronounced difference was observed for IgM detection (Z = − 3.00, *P* = 0.0027).

When comparing antibody classes, IgM detection showed significantly higher reactivity than IgG when analysed using ImageJ (Z = − 3.12, *P* = 0.0018), whereas no significant difference was observed between IgG and IgM using visual interpretation (*P* = 0.1936). These findings suggest that ImageJ-assisted analysis enhances the discrimination between antibody responses, which may not be apparent using conventional visual assessment. (Table 2, Table S4).

Binary logistic regression analyses were performed to evaluate whether IgG and IgM antibody detection using either visual inspection or ImageJ-assisted interpretation predicted opisthorchiasis. When IgM antibody detection was analysed using visual inspection and ImageJ interpretation, the overall regression model was statistically significant (χ² = 19.095, *P* = 0.0001), explaining approximately 59.9% of the variance in infection status (Nagelkerke R² = 0.599) (Table 2, Table S5). Although ImageJ-assisted IgM detection was associated with increased odds of infection (OR = 3.0), the predictor did not reach statistical significance (*P* = 0.466) (Table 2, Table S6). The model demonstrated good diagnostic discrimination (AUC = 0.832; 95% CI: 0.658–0.940) with an overall classification accuracy of 81.25% (Table 2, Table S5). In contrast, logistic regression analysis of IgG antibody detection showed weaker predictive performance. The overall model was statistically significant (χ² = 6.638, *P* = 0.036) but explained a smaller proportion of the variance (Nagelkerke R² = 0.250) (Table S5). IgG detection using visual inspection showed a trend towards association with infection (OR = 14.0, *P* = 0.055), whereas ImageJ-assisted interpretation was not associated with infection status (OR = 0.79, *P* = 0.808) (Table 2, Table S6). The model demonstrated moderate diagnostic discrimination (AUC = 0.699; 95% CI: 0.512–0.848) with an overall classification accuracy of 68.75% (Table 2, Table S5).

Further analyses incorporating both antibody classes demonstrated improved predictive performance. When IgG and IgM detection were analysed together using ImageJ-assisted interpretation, the regression model was statistically significant (χ² = 13.799, *P* = 0.001), explaining approximately 46.7% of the variance in diagnosis (Nagelkerke R² = 0.467) (Table 2, Table S5). In this model, IgM detection emerged as a significant predictor of infection (OR = 24.67, *P* = 0.006), whereas IgG detection remained non-significant (OR = 2.74, *P* = 0.266) (Table 2, Table S6). The model demonstrated good diagnostic discrimination (AUC = 0.842; 95% CI: 0.670–0.946) with an overall classification accuracy of 78.12% (Table 2, Table S5). When both antibodies were interpreted using visual inspection, the regression model showed the highest predictive performance (χ² = 23.643, *P* < 0.0001), explaining 69.6% of the variance in infection status (Nagelkerke R² = 0.696) and achieving excellent diagnostic discrimination (AUC = 0.895; 95% CI: 0.735–0.975) with an overall classification accuracy of 87.5% (Table 2, Table S5). IgG detection interpreted visually was identified as a significant predictor of infection (OR = 15.0, *P* = 0.040), whereas the extremely large odds ratio for IgM detection reflected quasi-complete separation of the data (Table 2, Table S6).

Overall, the optimised dot-ELISA demonstrated promising diagnostic potential for the serological detection of opisthorchiasis. The assay showed high sensitivity, particularly for IgM antibody detection, although specificity remained relatively limited due to cross-reactivity with other parasitic infections. The application of ImageJ-assisted analysis improved the objectivity and reproducibility of result interpretation compared with visual inspection alone. ROC analysis suggested that IgM detection provided better diagnostic discrimination than IgG, although the difference between antibody classes was not statistically significant. Additional statistical analyses, including agreement analysis, paired comparisons, and logistic regression modelling, further supported the diagnostic relevance of the assay, highlighting IgM antibody detection as the most important predictor of infection. When both antibody classes were analysed together, the predictive performance of the model improved, indicating that combined antibody detection may enhance the diagnostic capability of the dot-ELISA platform. Collectively, these findings suggest that the dot-ELISA system, particularly when combined with digital image analysis, represents a feasible and potentially useful approach for the serodiagnosis of opisthorchiasis.

### Stability of the recombinant protein coated on the NC membrane

The stability of the recombinant protein OvCB_OvAEP_OvCF coated on the NC was assessed by examining the reactivity of the dot-ELISA through visual inspection and ImageJ software.

**Fig. 2:**
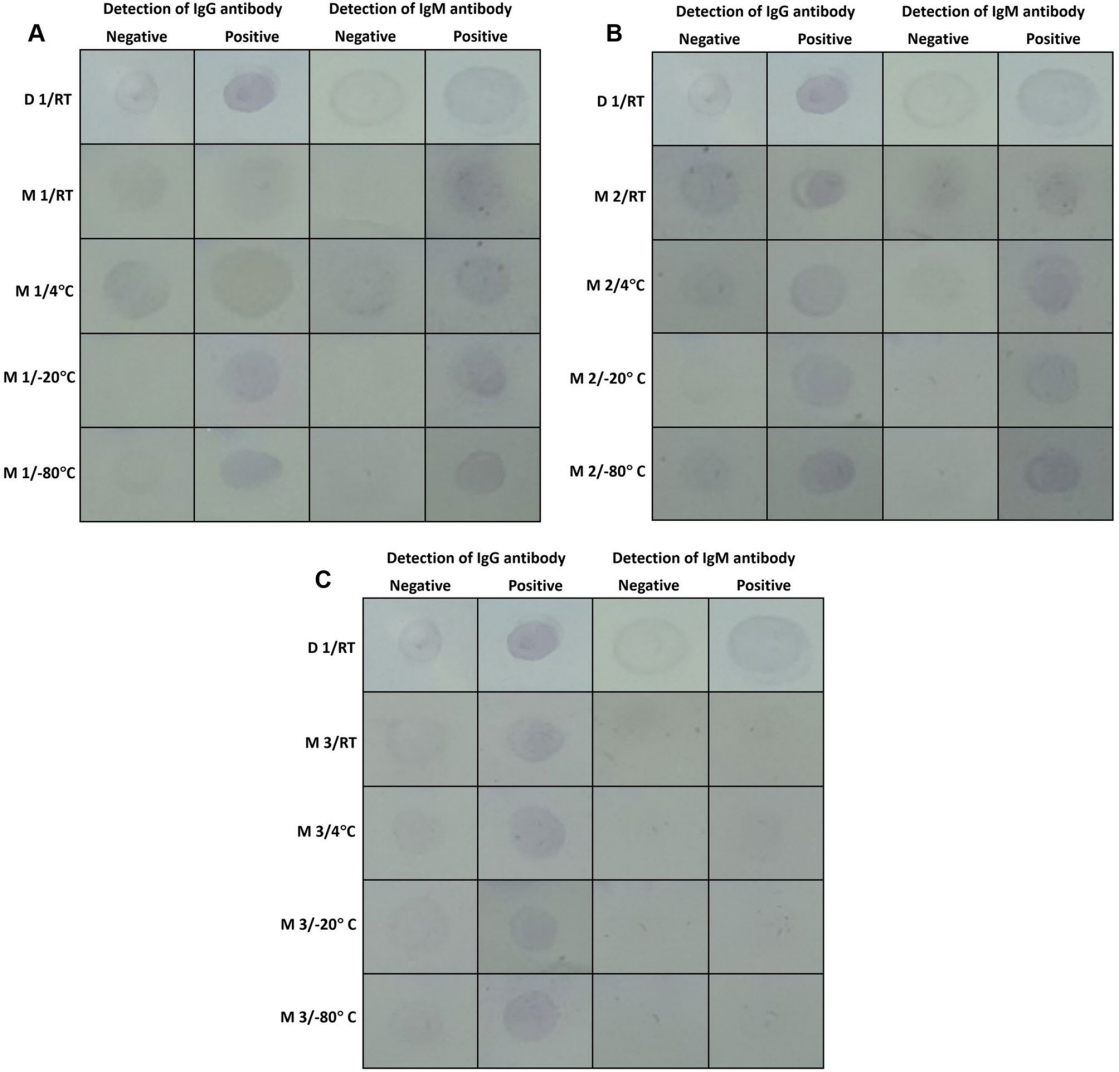
Stability evaluation of recombinant multi-epitope protein dotted on NC membranes under different storage conditions. Recombinant OvCB_OvAEP_OvCF protein dotted onto NC membranes stored at room temperature (25 °C), 4, −20, and −80 °C for (**A**) 1, (**B**) 2, and (**C**) 3 months. At each specified time point, membranes were subjected to dot-ELISA using pooled opisthorchiasis-positive serum and pooled negative serum to detect both IgG and IgM antibodies, to evaluate residual antigenicity and background reactivity, compared to the activity at day 1 (D1). Signal intensity and contrast between positive and negative reactions, evaluated using both visual inspection and ImageJ-assisted quantification, showed that storing protein at room temperature, 4, −20, and −80 °C for up to three months preserved its stability for IgG detection. The findings also showed that storage at all temperature points for up to two months preserved the protein stability needed for IgM detection

The denatured form of the recombinant protein could maintain its stability on NC up to three months at room temperature (25 °C), 4, −20, and −80 °C for detecting IgG antibody. Under these conditions, a positive signal was clearly distinguished from the reactivity signals of the pooled negative serum, which was similar to the reactivity on the first day (D1). However, the signal from the pool of negative sera varied with some high background values, which may indicate nonspecific binding or background staining (Fig. 2).

For the detection of IgM antibodies, the recombinant protein-coated NC strips stored at room temperature (25 °C), 4, −20, and −80 °C for one and two months exhibited a strong reactivity signal with positive pool serum. Under these conditions, the negative signals varied, exhibiting some instances of high background levels. However, the positive signals exhibited significantly higher integrated colour intensity compared to the negative signals (data not shown). This difference in intensity allowed the discrimination between positive and negative results through analysis using the ImageJ software and visual inspection. Nonetheless, extended storage for up to three months at all temperatures did not result in positive signals, suggesting a potential loss of antigen reactivity for IgM detection (Fig. 2).

## Discussion

Accurate and accessible diagnostic tools are essential for the control of opisthorchiasis in endemic regions, where infection remains highly prevalent and is strongly associated with CCA. In the present study, we developed and evaluated an in-house dot-ELISA platform based on a multi-epitope recombinant antigen, OvCB_OvAEP_OvCF, for the serological detection of OV. Overall, the assay demonstrated high sensitivity, particularly for IgM antibody detection, while the incorporation of ImageJ-assisted digital analysis improved the objectivity and reproducibility of result interpretation. These findings support the potential of combining recombinant antigen technology with digital image analysis to enhance the diagnostic utility of serological assays for opisthorchiasis.

A key methodological strength of this study was the quantitative evaluation of dot-ELISA signals using raw integrated density measurements obtained from ImageJ software. This parameter incorporates both pixel intensity and spot area, thereby reflecting the overall chromogenic signal generated by antigen–antibody interactions. This is particularly relevant in dot-ELISA, where slight variation in spot diameter may arise from manual spotting or membrane absorption. In addition, the use of ROC-derived cut-off values provided an objective threshold for distinguishing positive from negative reactions, improving analytical robustness compared with simple qualitative grading of dot intensity. Together, these approaches enhanced methodological transparency and supported reproducibility across different settings.

The extended statistical analyses provided further insight into the diagnostic behaviour of the assay. ROC analysis demonstrated moderate discriminatory ability for IgM antibody detection, whereas IgG detection showed lower performance. Although the difference between IgM and IgG ROC curves was not statistically significant, logistic regression modelling identified IgM detection as the strongest predictor of OV infection, consistent with its role in early immune responses. Agreement analysis further showed substantial concordance between visual inspection and ImageJ-assisted interpretation for IgM detection and moderate agreement for IgG detection, indicating that digital analysis improves consistency, particularly in borderline cases.

Importantly, the Wilcoxon signed-rank test provided complementary evidence supporting the added value of ImageJ-assisted interpretation. Significant differences were observed between visual and ImageJ-based classification, particularly for IgG detection (*P* < 0.0001), suggesting that visual interpretation may overestimate positivity, likely due to subjective assessment of weak signals. A similar, though less pronounced, effect was observed for IgM detection (*P* = 0.0027). Notably, ImageJ-assisted analysis enabled clearer differentiation between IgG and IgM responses (*P* = 0.0018), whereas no significant difference was detected using visual interpretation (*P* = 0.1936). This finding indicates that conventional visual assessment may mask biologically meaningful differences between antibody classes and highlights the importance of semi-quantitative image analysis in improving diagnostic discrimination and reducing observer bias.

One of the most notable findings of this study was the strong diagnostic contribution of IgM antibody detection. IgM detection achieved very high sensitivity, reaching 100% by visual inspection and 93.33% using ImageJ analysis, whereas IgG detection showed more moderate sensitivity and specificity. These observations are consistent with the immunological characteristics of helminth infections, in which IgM antibodies usually reflect early immune responses after parasite exposure, whereas IgG antibodies may persist for longer periods and therefore represent both past and current infection. This suggests that IgM detection may offer particular value for identifying recent or active infection, while IgG responses may reflect cumulative exposure within endemic populations. However, combining IgM and IgG detection of dot-ELISA in the present study may improve overall screening sensitivity, as individual patients may exhibit different antibody profiles depending on the stage of infection and host immune response.

Despite the high sensitivity observed for IgM detection, the assay showed relatively low specificity owing to cross-reactive antibody responses. Cross-reactivity was particularly evident in sera from patients with other parasitic infections, including strongyloidiasis, taeniasis, hookworm infection, giardiasis, and *E. coli* infection. Such cross-reactivity is likely to reduce the specificity of the assay and may limit its diagnostic value in co-endemic regions. These findings are consistent with previous studies showing that OV serological assays may have reduced specificity in settings where polyparasitism is common and where shared epitopes among co-endemic parasites complicate serological discrimination during both acute and chronic phases of infection [3, 4, 15].

Within the broader diagnostic landscape of opisthorchiasis, the present dot-ELISA should therefore be interpreted as a screening or triage tool rather than a standalone confirmatory assay. The moderate positive predictive values observed in this study suggest that positive results should be interpreted cautiously and, where appropriate, confirmed using complementary diagnostic approaches. Conventional stool examination methods remain widely used but are limited by poor sensitivity in light infections and the need for repeated faecal sampling. Molecular techniques such as PCR provide high analytical sensitivity for detecting parasite DNA but require specialised laboratory infrastructure and trained personnel. Serological methods, including ELISA and immunoblotting, have improved sensitivity for detecting low-intensity infections but may require laboratory equipment and proprietary reagents [16]. Immunochromatographic tests (ICTs) have also shown satisfactory sensitivity and specificity in detecting both antigens and antibodies for opisthorchiasis diagnosis, but their application remains limited by visual interpretation and, in some cases, restricted to IgG-based detection [17–21]. In comparison, the simplified dot-ELISA platform developed in this study offers several operational advantages, including minimal instrumentation requirements, rapid assay processing, and reduced reagent consumption, making it particularly suitable for screening applications in resource-limited settings. Additionally, the dot-ELISA platform developed in this study detects both IgM and IgG. Combined with ImageJ-assisted quantification, it provides diagnostic coverage, improves individual identification across heterogeneous infection stages in endemic populations, and reduces observer-dependent bias, especially in samples that are borderline or weakly positive.

The use of a multi-epitope recombinant antigen represents an additional strength of the present study. By combining multiple B-cell epitopes derived from cathepsin B, cathepsin F, and asparaginyl endopeptidase into a single recombinant construct, the OvCB_OvAEP_OvCF antigen may enhance antibody recognition across heterogeneous host immune responses. Furthermore, the denatured form of the recombinant protein used in this study may have disrupted its three-dimensional conformation and exposed linear epitopes that were previously concealed within the native structure. This may have facilitated stronger recognition by antibodies. In this context, the denatured OvCB_OvAEP_OvCF recombinant protein may act as a particularly useful early-detection antigen for opisthorchiasis. In addition, this denatured form appears to bind efficiently to nitrocellulose membranes, which may further support its utility in dot-based immunoassays [22].

From a public health perspective, improved diagnostic tools remain essential for the control of opisthorchiasis in Southeast Asia, particularly within the Mekong Basin, where infection prevalence remains high. Chronic OV infection is strongly associated with CCA, one of the most important infection-related cancers in the region. Early detection and treatment are therefore critical components of disease control and cancer prevention strategies. The dot-ELISA platform developed in this study may provide a practical screening tool for community-based surveillance programmes, especially in rural areas where laboratory infrastructure is limited. Within a broader One Health framework, improved diagnostic capacity may also support integrated control strategies targeting parasite transmission at the interface of human, animal, and environmental reservoirs.

Another important strength of the proposed platform is its operational robustness. The recombinant antigen immobilised on nitrocellulose membranes maintained detectable reactivity for up to three months under several storage conditions for IgG detection and up to two months for IgM detection. Importantly, stability at room temperature suggests that the assay could be deployed without dependence on cold-chain logistics, which represents a significant practical advantage for diagnostic use in remote or resource-limited areas.

Moreover, the integration of digital image capture and ImageJ-assisted analysis provides additional practical benefits. Dot-ELISA results can be photographed using smartphone cameras and analysed with open-source software, facilitating documentation, retrospective review, remote validation, and data sharing. This may improve standardisation across different observers and study settings and could support decentralised diagnostic strategies and tele-diagnostic applications in large-scale screening programmes.

Several limitations should nevertheless be considered. First, the relatively small sample size and lack of representation of acute, chronic, and post-treatment cases, which may have affected the precision of the estimated diagnostic parameters. Secondly, cross-reactivity with other parasitic infections reduced assay specificity. Future studies should therefore focus on refining epitope design to minimise cross-reactive responses. Strategies such as in silico prediction and targeted removal of cross-reactive epitopes, or selective truncation of conserved protease domains, may further improve diagnostic specificity without substantially compromising sensitivity. In addition, validation in larger populations across multiple endemic regions will be necessary. The incorporation of the multi-epitope antigen into alternative diagnostic formats, such as lateral flow assays, may also facilitate the development of rapid point-of-care tools.

Overall, the findings of this study demonstrate that the combination of a multi-epitope recombinant antigen with a dot-ELISA platform and digital image analysis represents a promising strategy for improving the serological diagnosis of opisthorchiasis, particularly for screening applications in endemic and resource-limited settings.

## Conclusion

In conclusion, the dot-ELISA platform developed in this study, based on the multi-epitope recombinant antigen OvCB_OvAEP_OvCF, demonstrated promising performance for the serological detection of opisthorchiasis. The assay achieved high sensitivity, particularly for IgM antibody detection, while ImageJ-assisted analysis enhanced the objectivity, reproducibility, and diagnostic consistency of result interpretation. Importantly, complementary statistical analyses, including Wilcoxon signed-rank testing, demonstrated that ImageJ-assisted quantification significantly improved classification accuracy and enabled clearer differentiation between IgG and IgM responses, which may not be reliably distinguished using visual interpretation alone. Despite these strengths, specificity was limited by cross-reactive antibody responses in co-endemic parasitic infections, indicating that the assay is better suited as a screening or triage tool rather than a standalone confirmatory diagnostic method. The combined assessment of IgG and IgM responses further improved diagnostic utility across different stages of infection. In addition, the demonstrated stability of antigen-coated membranes under various storage conditions, including at room temperature, supports the feasibility of deploying this platform in field-based and resource-limited settings. With further optimisation to reduce cross-reactivity and validation in larger and more diverse populations, this dot-ELISA system, particularly when integrated with semi-quantitative digital analysis, may provide a practical, scalable, and standardised approach for surveillance and early detection of opisthorchiasis in endemic regions. 

## Supplementary Information

Below is the link to the electronic supplementary material.

Supplementary Material 1

## Data Availability

All data generated or analysed during this study are included in this published article and its supplementary information files.

## Abbreviations

OV: *Opisthorchis viverrini*
ELISA: Enzyme-linked immunosorbent assay
RDT: Rapid diagnostic tests
m-FECT: Modified formalin-ether concentration technique
ICT: Immunochromatographic test
CCA: Cholangiocarcinoma
NC: Nitrocellulose
LB: Luria–Bertani
Ni-NTA: Nickel-nitrilotriacetic acid
HRP: Horseradish peroxidase
TMB: 3,3’,5,5’-tetramethyl benzidine
BSA: Bovine serum albumin
PBST: Phosphate-buffered saline with tween
ROC: Receiver operating characteristic
AUC: Area under the curve
