# Supplementary material for: Enhanced serodiagnosis of opisthorchiasis using a multi-epitope dot-ELISA: comparative evaluation of visual and imageJ-assisted analysis of IgG and IgM responses
Source: BMC Infect Dis. 2026 Apr 29;26:1154. doi: 10.1186/s12879-026-13445-w (PMC13274237; doi:10.1186/s12879-026-13445-w)
Supplement: Supplementary file 1 — Supplementary Material 1 [file 12879_2026_13445_MOESM1_ESM.docx]

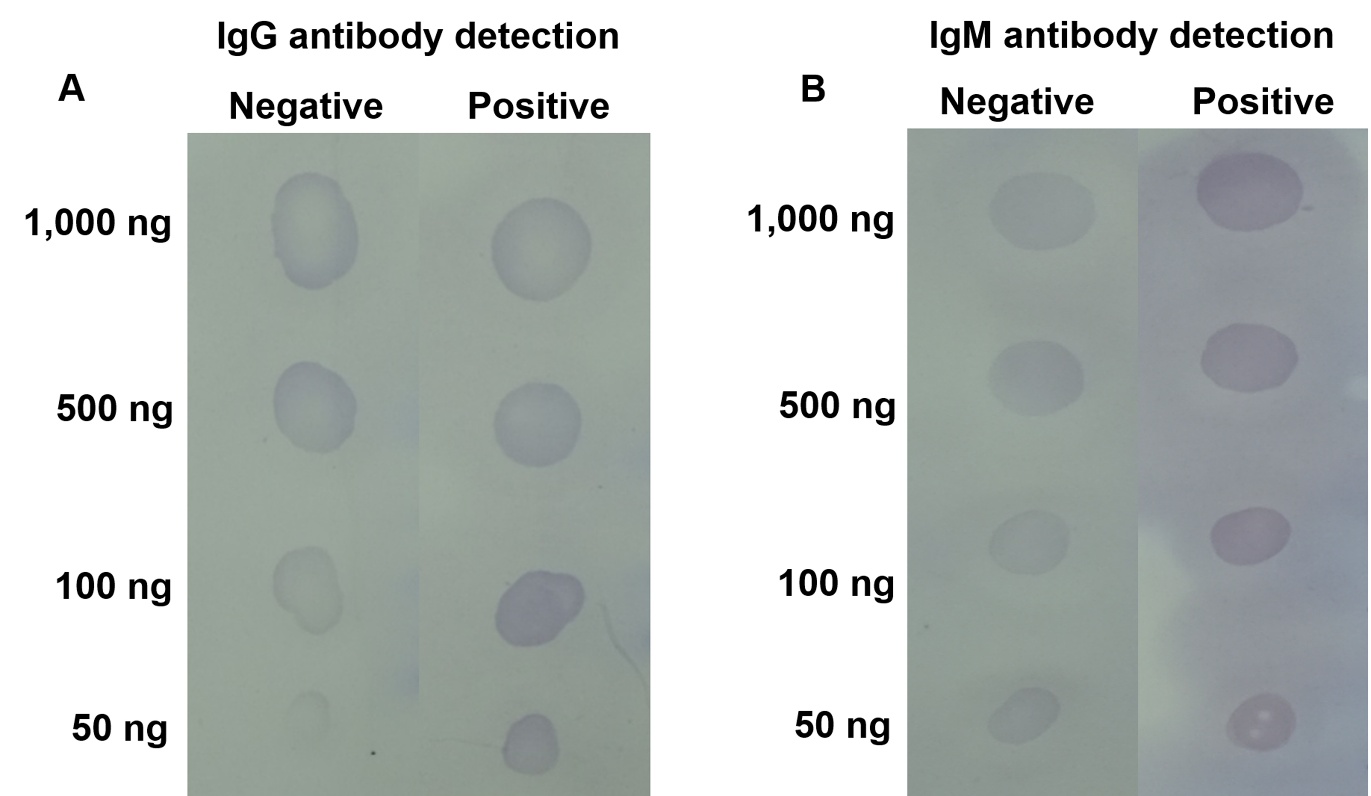


**Figure S1**. Optimisation of dot-ELISA conditions for IgG and IgM antibody detection using pooled sera. The optimal assay conditions were determined using pooled sera from OV-positive and -negative individuals. For IgG antibody detection (A), optimal conditions included a recombinant antigen concentration of 50 ng/µL, a human serum dilution of 1:2,000, and a goat anti-human IgG-HRP conjugate dilution of 1:5,000. For IgM antibody detection (B), optimal conditions were established using a recombinant antigen concentration of 100 ng/µL, a human serum dilution of 1:1,000, and a goat anti-human IgM-HRP conjugate dilution of 1:5,000. These conditions were selected based on optimal signal-to-background discrimination, as determined by concordant visual assessment and ImageJ-assisted signal quantification.


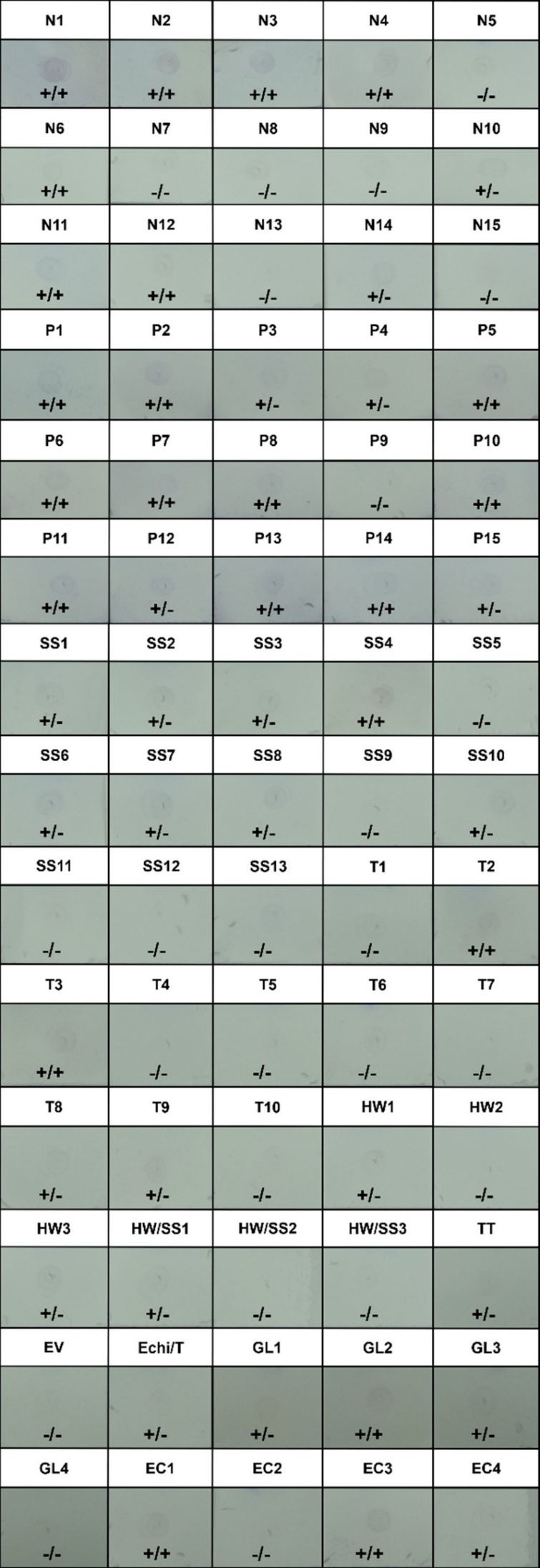


**Figure S2** IgG antibody detection by dot-ELISA using sera from individuals with various parasitic infections and uninfected controls. Dot-ELISA results for anti-OvCB_OvAEP_OvCF IgG antibody detection are shown for individual serum samples. Results are presented as paired outputs, with the qualitative interpretation by visual inspection shown before the forward slash ( / ) and the corresponding ImageJ-assisted classification shown after the slash, based on cut-off values derived from the optimised assay. In both approaches, “+” indicates positive reactivity and “–” indicates negative reactivity. Tested sera were from parasitologically confirmed cases of *Opisthorchis viverrini* infection (P), strongyloidiasis (SS), taeniasis (T), hookworm infection (HW), mixed *S. stercoralis* and hookworm infection (HW/SS), trichuriasis (TT), enterobiasis (EV), mixed *Echinostoma* spp. and *Taenia* spp. infection (Echi/T), giardiasis (GL), and *E. coli* infection (EC), along with uninfected controls (N).


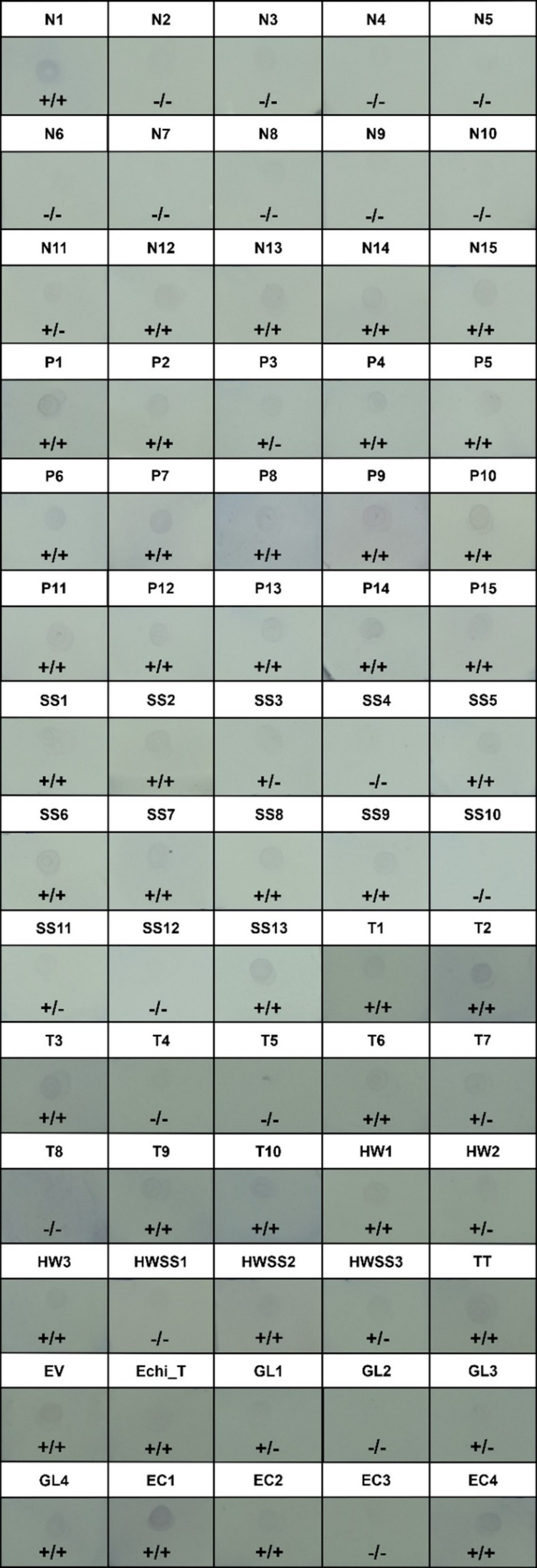


**Figure S3.** IgM antibody detection by dot-ELISA using sera from individuals with various parasitic infections and uninfected controls. Dot-ELISA results for anti-OvCB_OvAEP_OvCF IgM antibody detection are shown for individual serum samples. Results are presented as paired outputs, with qualitative interpretation by visual inspection shown before the forward slash ( / ) and ImageJ-assisted classification shown after the slash, based on cut-off values derived from the optimised assay. In both approaches, “+” indicates positive reactivity and “–” indicates negative reactivity. Sera included parasitologically confirmed infections: *Opisthorchis viverrini* (P), strongyloidiasis (SS), taeniasis (T), hookworm infection (HW), mixed *S. stercoralis* and hookworm infection (HW/SS), trichuriasis (TT), enterobiasis (EV), mixed *Echinostoma* spp. and *Taenia* spp. infection (Echi/T), giardiasis (GL), and *E. coli* infection (EC), together with negative controls (N).

**Table S1** Comparison of ROC curves between IgG and IgM detection using ImageJ-assisted interpretation.

| **Comparison** | **Difference in AUC** | **SE** | **z-statistic** | **P-value** |
| --- | --- | --- | --- | --- |
| IgG vs IgM | −0.219 | 0.1339 | −1.636 | 0.1019 |

| **Comparison** | **Cohen’s Kappa** | **Standard error** | **95% CI** | **Interpretation** |
| --- | --- | --- | --- | --- |
| IgG Visual vs ImageJ | 0.449 | 0.084 | 0.28497 to 0.61391 | Moderate agreement |
| IgM Visual vs ImageJ | 0.711 | 0.0862 | 0.54187 to 0.87962 | Substantial agreement |
| IgG vs IgM (Visual) | 0.038 | 0.118 | -0.19254 to 0.26837 | Slight agreement |
| IgG vs IgM (ImageJ) | 0.014 | 0.103 | -0.18778 to 0.21520 | Slight agreement |

**Table S2** Agreement between antibody detection methods and interpretation approaches was assessed using Cohen’s kappa coefficient.

**Table S3** Paired comparison of diagnostic classification using McNemar’s test. McNemar’s test was used to compare paired diagnostic classifications between visual inspection and ImageJ-assisted interpretation for IgG and IgM antibody detection, as well as between antibody classes under the same interpretation mode. A P-value < 0.05 was considered statistically significant.

| **Comparison** | **Difference in proportion** | **95% CI** | **P-value** |
| --- | --- | --- | --- |
| IgG Visual vs ImageJ | 0.3000 | 19.26% to 40.74% | <0.0001 |
| IgM Visual vs ImageJ | 0.1286 | 5.02% to 20.70% | 0.0039 |
| IgG vs IgM (Visual) | 0.1000 | −4.90% to 24.90% | 0.2649 |
| IgG vs IgM (ImageJ) | 0.2714 | 11.34% to 42.94% | 0.0026 |

**Table S4** Comparison of diagnostic outcomes using the Wilcoxon signed-rank test. The Wilcoxon signed-rank test was used to assess paired differences between interpretation methods (visual inspection vs ImageJ-assisted analysis) and between antibody classes (IgG vs IgM). Results were presented as median values, and Z statistics. A two-tailed significance level of P-value < 0.05 was considered statistically significant. ImageJ-assisted analysis significantly altered classification outcomes compared with visual interpretation, particularly for IgG detection (P < 0.0001). Furthermore, IgM responses were significantly higher than IgG responses when analysed using ImageJ (P = 0.0018), whereas no significant difference was observed using visual interpretation (P = 0.1936). These findings indicate that ImageJ-assisted analysis improves diagnostic discrimination and reduces observer-dependent bias.

| **Comparison** | **Median (Group 1)** | **Median (Group 2)** | **Z value** | **P-value** | **Interpretation** |
| --- | --- | --- | --- | --- | --- |
| IgG (ImageJ vs Visual) | 0 | 1 | −4.58 | < 0.0001 | Significant difference; visual interpretation overestimates positivity |
| IgM (ImageJ vs Visual) | 1 | 1 | −3.00 | 0.0027 | Significant difference; ImageJ refines classification |
| IgG vs IgM (ImageJ) | 0 | 1 | −3.12 | 0.0018 | Significant difference; IgM shows higher reactivity |
| IgG vs IgM (Visual) | 1 | 1 | −1.30* | 0.1936 | No significant difference |

*Z value approximated from P-value (if not explicitly reported)

**Table S5** Model performance statistics of logistic regression models for predicting opisthorchiasis. Model fit and predictive performance of the logistic regression models evaluating IgG and IgM antibody detection using visual inspection and ImageJ-assisted interpretation. Model statistics include the likelihood ratio chi-square test (χ²) for overall model significance, Nagelkerke R² indicating the proportion of variance explained by the model, the area under the receiver operating characteristic curve (AUC) representing diagnostic discrimination, and the overall classification accuracy of each model.

| **Model** | **χ²** | **P-value** | **Nagelkerke R²** | **AUC** | **Accuracy** |
| --- | --- | --- | --- | --- | --- |
| IgG model (Visual vs ImageJ) | 6.638 | 0.036 | 0.250 | 0.699 | 68.75% |
| IgM model (Visual vs ImageJ) | 19.095 | 0.0001 | 0.599 | 0.832 | 81.25% |
| Combined ImageJ model (IgG and IgM) | 13.799 | 0.001 | 0.467 | 0.842 | 78.12% |
| Combined Visual model (IgG and IgM) | 23.643 | <0.0001 | 0.696 | 0.895 | 87.50% |

**Table S6** Binary logistic regression analysis of IgG and IgM antibody detection for predicting opisthorchiasis. Binary logistic regression models were constructed to evaluate the predictive value of IgG and IgM antibody detection interpreted by visual inspection and ImageJ-assisted analysis. Separate models were generated to compare interpretation methods for each antibody class, and combined models including both antibodies under the same interpretation approach. Regression coefficients (β), odds ratios (OR), 95% confidence intervals (CI), and P-values are presented for each predictor. Extremely large odds ratios occurred in some predictors due to quasi-complete separation of the data.

| **Model** | **Predictor** | **Coefficient (β)** | **Odds ratio (OR)** | **95% CI** | **P-value** |
| --- | --- | --- | --- | --- | --- |
| IgG model (Visual vs ImageJ) | IgG_ImageJ | −0.241 | 0.79 | 0.11–5.49 | 0.808 |
|  | IgG_Visual | 2.639 | 14.00 | 0.94–207.61 | 0.055 |
| IgM model (Visual vs ImageJ) | IgM_ImageJ | 1.099 | 3.00 | 0.16–57.37 | 0.466 |
|  | IgM_Visual | 21.505 | extremely large* | – | 0.998 |
| Combined model (ImageJ) | IgG_ImageJ | 1.009 | 2.74 | 0.46–16.22 | 0.266 |
|  | IgM_ImageJ | 3.205 | 24.67 | 2.46–247.20 | 0.006 |
| Combined model (Visual) | IgG_Visual | 2.708 | 15.00 | 1.14–198.05 | 0.040 |
|  | IgM_Visual | 22.420 | extremely large* | – | 0.998 |
